# Supplementary material for: Identification of Freezing-Responsive microRNAs and Their Targets in Chinese Jujube by Small RNA and Degradome Sequencing
Source: Int J Mol Sci. 2026 Sep 4;27(17):7912. doi: 10.3390/ijms27177912 (PMC13566890; doi:10.3390/ijms27177912)
Supplement: Supplementary file 1 [file ijms-27-07912-s001.zip › Supplementary Material S2/novel_miR_89.pdf]

Provisional ID : novel_miR_89
Score total : 24.1
Score for star read(s) : 3.9
Score for read counts : 21.5
Score for mfe : 0.9
Score for randfold : -2.2
Score for cons. seed :
Total read count : 54
Mature read count : 50
Loop read count : 3
Star read count : 1

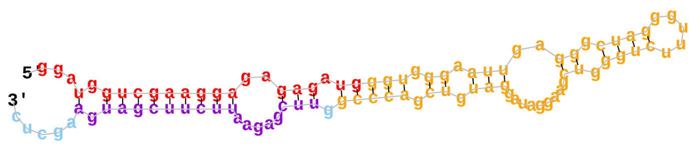

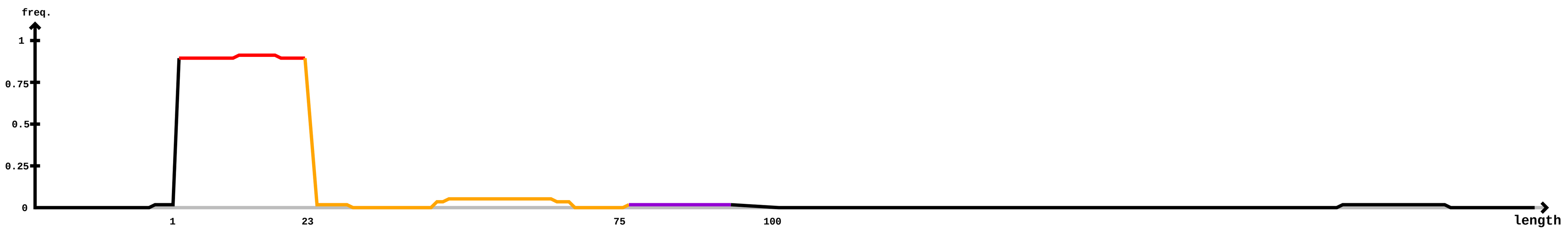

Mature Star

```
5'- caugagcuugguggguggguaaggauggucgaaggagagagaugggugggaauugagggcuaggguuucugggucgaaggauaggaugucgacccgguucgagaauucuucgaugaagcucccgacggcgauucggugguggcgcguucgugccggugaggaggaggacaaggccauggcguauaaaggauaaggggauucauaagucaccgaauuaucggaucugagaugagauuccugcgucgaauuu -3' obs
    caugagcuugguggguggguaaggauggucgaaggagagagaugggugggaauugagggcuaggguuucugggucgaaggauaggaugucgacccgguucgagaauucuucgaugaagcucccgacggcgauucggugguggcgcguucgugccggugaggaggaggacaaggccauggcguauaaaggauaaggggauucauaagucaccgaauuaucggaucugagaugagauuccugcgucgaauuu     exp
    ..((((.(((.((..((((..((..(.(((((((((..(((.(((((.((.(((..((.((((.....)))).)).........))).)).))))).))).....)))))))).)..))))))).)).)))))))((.(((((..((((...((.....))....))))...))))).)).......(((..((((((((((...(((((((....))))...))).)))).)))))))..)))......     reads  mm  sample
    ..................guaaggauggucgaaggagag...................................................................................................................................................................................................................     1      0   LT-1
    ......................ggauggucgaaggagagagaug..............................................................................................................................................................................................................     25     0   LCK-2
    ......................ggauggucgaaggagagagaug..............................................................................................................................................................................................................     25     0   LCK-1
    ................................aggaAagagaugggugga........................................................................................................................................................................................................     1      1   JT-3
    .................................................................uuucugggucgaaggauaggaug..................................................................................................................................................................     2      0   LT-2
    ...................................................................ucugggucgaaggauagg.....................................................................................................................................................................     1      0   LT-3
    .................................................................................................uucgagaauucuucAauga......................................................................................................................................     1      1   JT-1
    .......................................................................................................................................................................................................................aucggaucugagaugaga.................     1      0   LT-1
```
